# The misdiagnose and treatment of a concealed kind of supernumerary nostril: a case report and review

**DOI:** 10.1186/s12893-022-01679-9

**Published:** 2022-06-13

**Authors:** Caiyun Li, Qianqian Pang, Haiying Zhou, Ahmad Alhaskawi, Yanzhao Dong, Hui Lu

**Affiliations:** 1 Department of Plastic Surgery, The First Affiliated Hospital, College of Medicine, Zhejiang University, # 79Qingchun Road, Zhejiang 310003 Hangzhou, People’s Republic of China; 2 Department of Orthopedics, The First Affiliated Hospital, College of Medicine, Zhejiang University, # 79Qingchun Road, Zhejiang 310003 Hangzhou, People’s Republic of China

**Keywords:** Congenital nasal malformation, Supernumerary nostril, Nasal sinus, Nasal dermal sinus, Misdiagnosis

## Abstract

**Background:**

Supernumerary Nostril, also called triple nostrils or accessory nostril, is a rare congenital nasal malformation.

**Case presentation:**

We report one conceal case of supernumerary nostril in a 19-years-old men which is misdiagnosed to a simple small nasal skin pit. Ordinary surgical excision led to recurrent infection of the lesion postoperatively, and was eventually required secondary surgery and the lesion was finally confirmed by pathological biopsy as a trinasal nostrils.

**Conclusions:**

Through this case, we stress the essential role in differential diagnosis, confirming the diagnosis and seeking for better solutions.

*Level of Evidence* V

## Background

Supernumerary nostril is a rare congenital defect, with approximately 60 related cases reported since Lindsay was first reported in 1906 [1]. In some reported cases, the patients appear to have an accessory nostril, either above or below the normal nostrils, which can be easily diagnosed. However, we report a misdiagnosed case of unobvious congenital supernumerary nostril, which to our knowledge, no similar cases have been reported.

## Case report

**Fig. 1:**
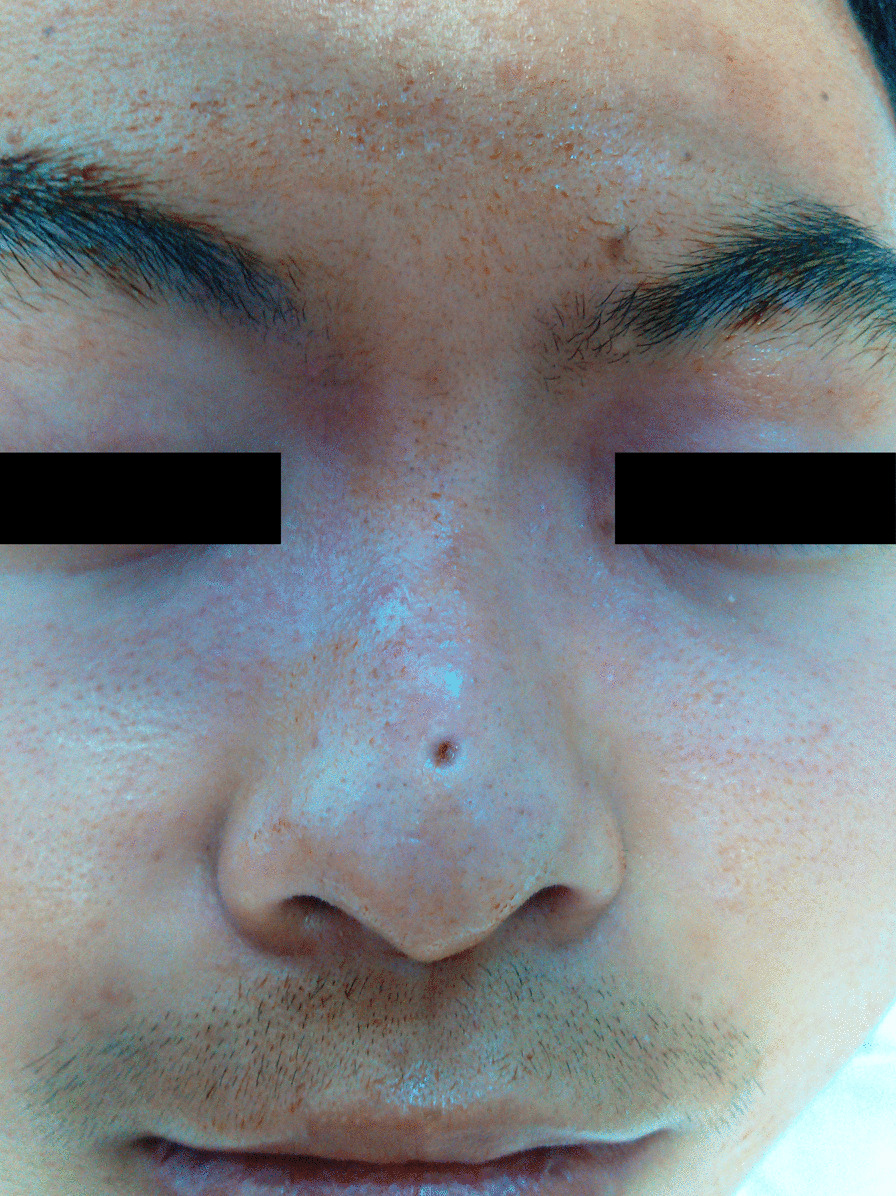
Patient with a small pit on the nasal dorsum before the first operation

**Fig. 2:**
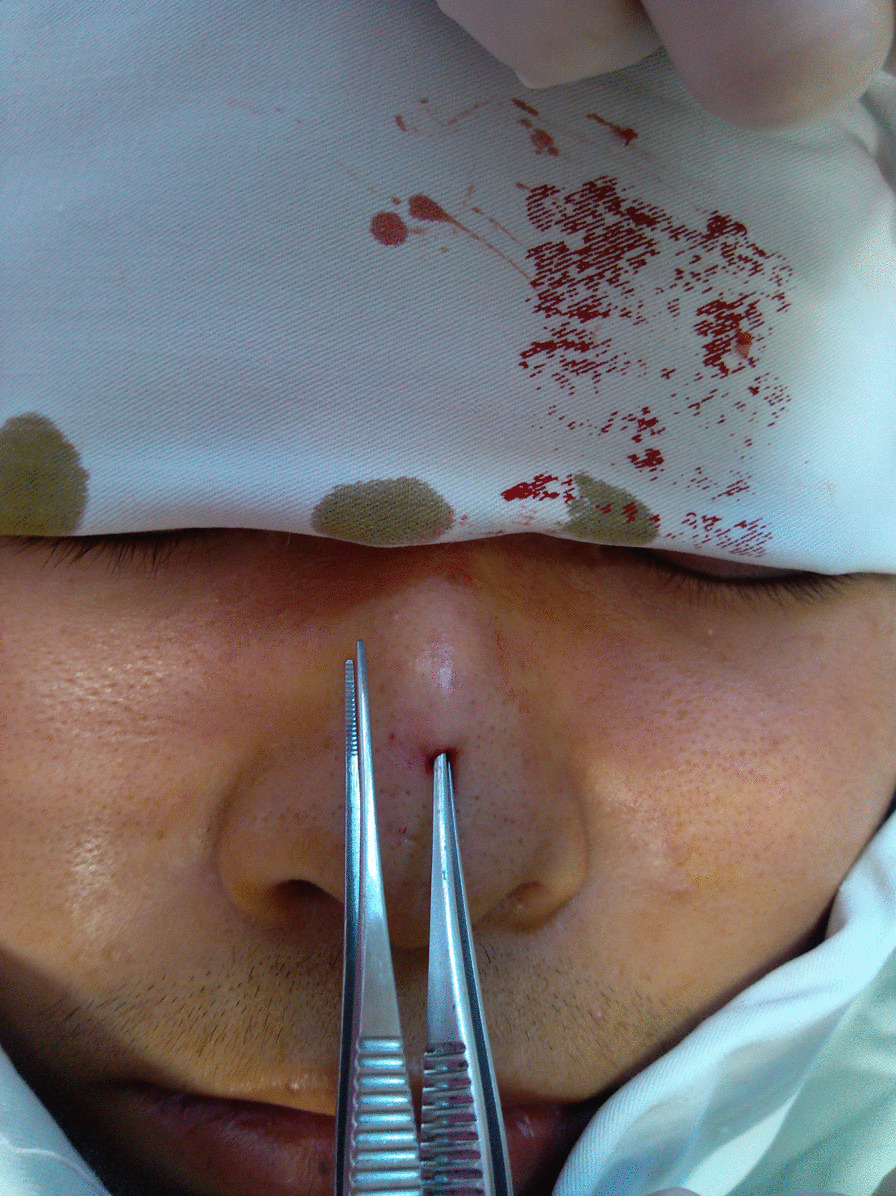
There was a sinus paralleling to the nasal dorsum which was about 2 cm in length

A 19-year-old man came to our department because of a small pit on the nasal dorsum which made him look different (Fig. 1). The small pit ranged 3 mm in diameter was found when he was born and had not increase in size significantly, nor discovered fluid or pus but a few sebaceous like secretion. There are no similar diseases in the family. It was diagnosed as a simple skin pit malformation. So, we decided to remove the pit. During the operation of the skin pit resection, we found that there was a sinus paralleling to the nasal dorsum which was about 2 cm in length (Fig. 2), but the patient refused further surgery and the incision was sutured as a palliative.

Within 1-year post surgery, the incision area was repeatedly infected with subcutaneous abscess. CT scan with contrast medium infused into the sinus showed that there was no obvious deformity in the nasal bone and skull base. The diameter of the sinus is about 3 mm wide and its depth is about 2 cm. And, the sinus and the nasal cavity are not connected (Gif.1). We concluded with the diagnosis of a supernumerary nostril.

**Fig. 3:**
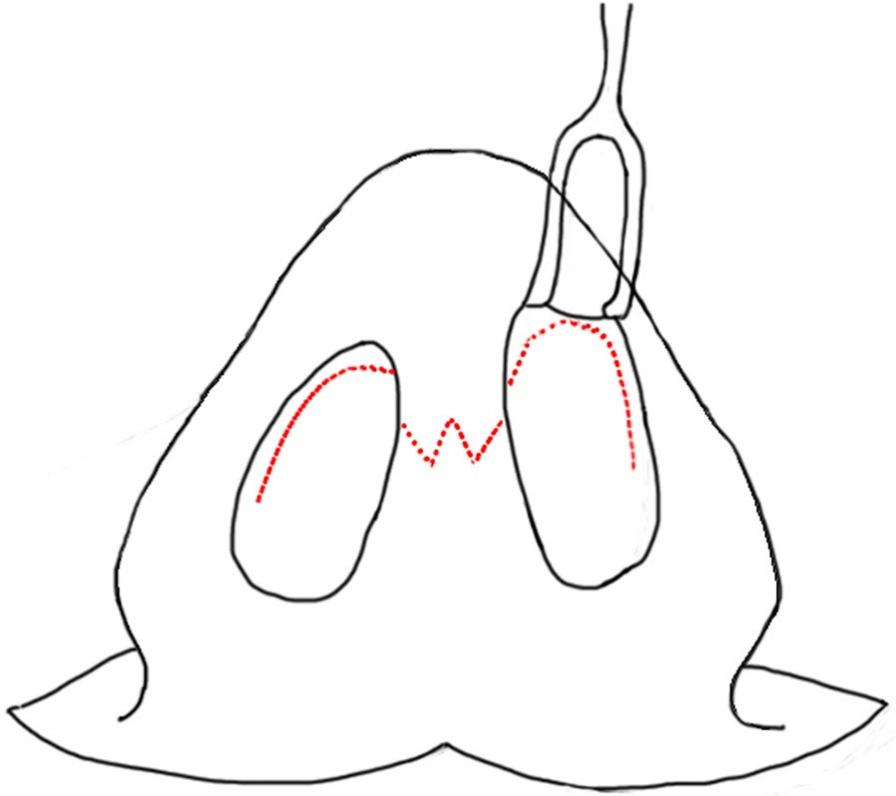
An Inverted-V shaped incision was designed

**Fig. 4:**
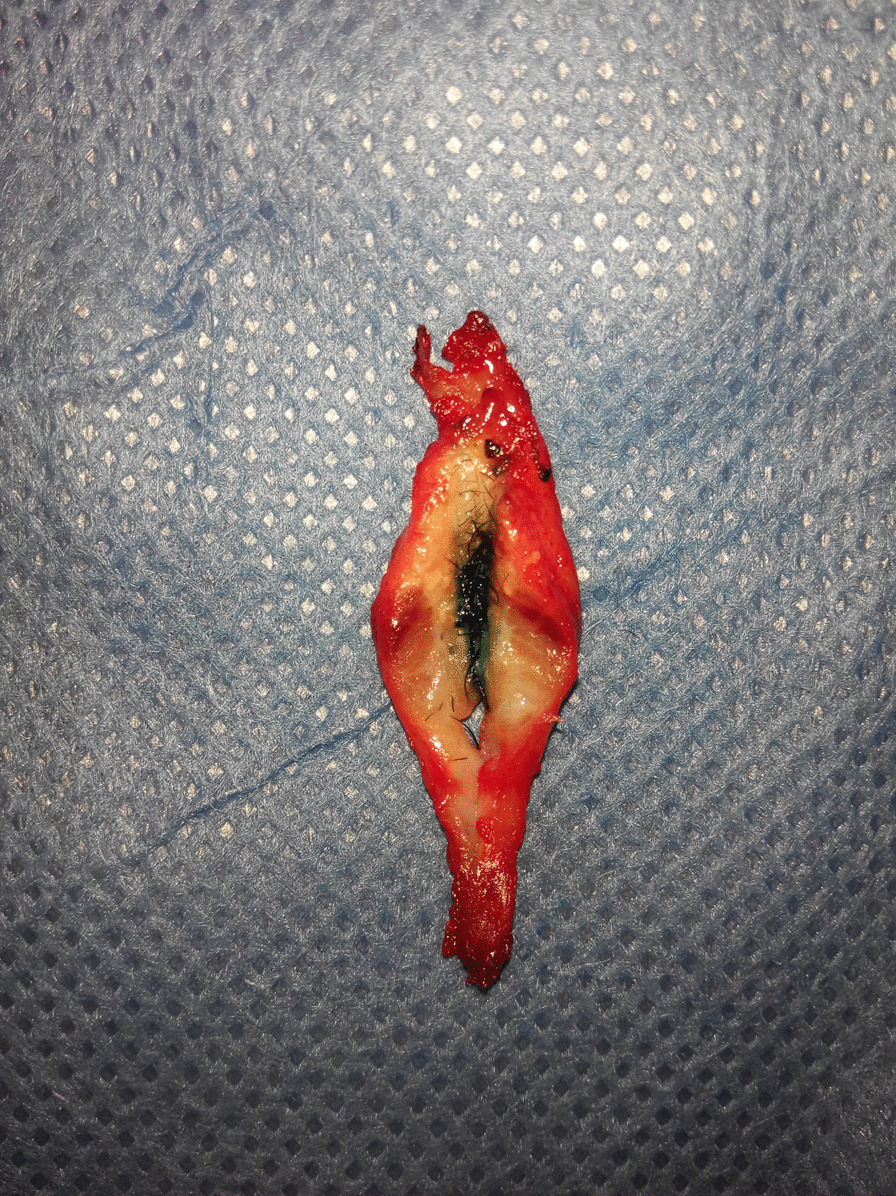
The showing of the removed sinus cutted: open:methylene blue show it was a blind sinus and there were thick rhinothrix

**Fig. 5:**
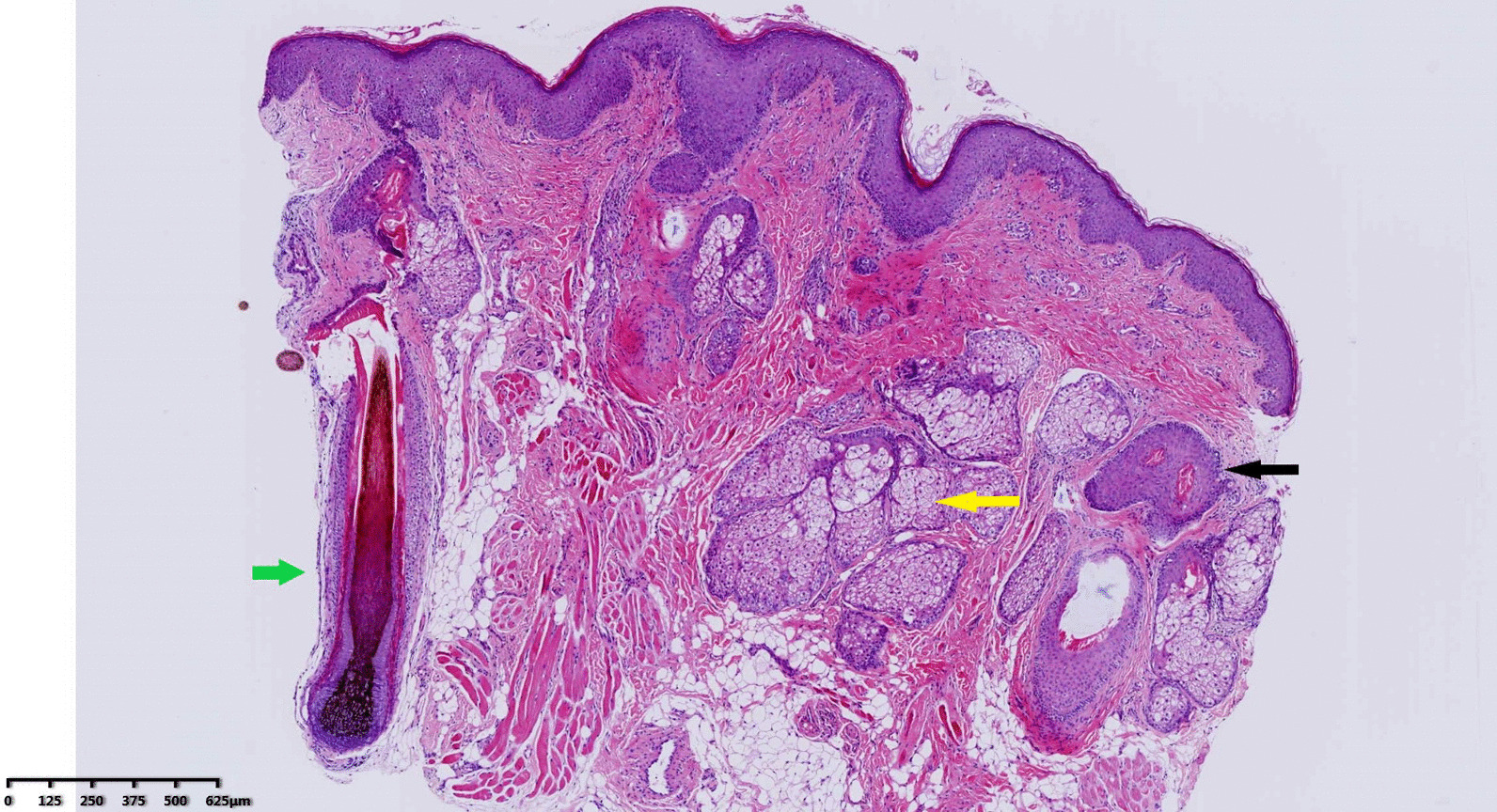
Histology showed hairy root (green arrow), hair follicles (black arrow) and rich sebaceous glands (yellow arrow)

Design: The surgery was designed with an Inverted-V shaped incision on the columella and extended to the nasal cavity along the lower edge of the alar cartilage (Fig. 3). The skin was cut open, then dissected upward and separated the sinus between the skin and bone. We are able to see the mucosa or skin like structure in the sinus and the thick, black nose hair evenly distributed. We verify the nasal cavity is not connected to the sinus, and we removed the accessory sinus nostril completely. The size of the accessory mass is about 2 cm × 3 mm (Fig. 4); and the excess composite tissue is trimmed. Then the skin incision was sutured and bandaged with pressure. Histology showed hairy root, hair follicles and rich sebaceous glands (Fig. 5).

**Fig. 6:**
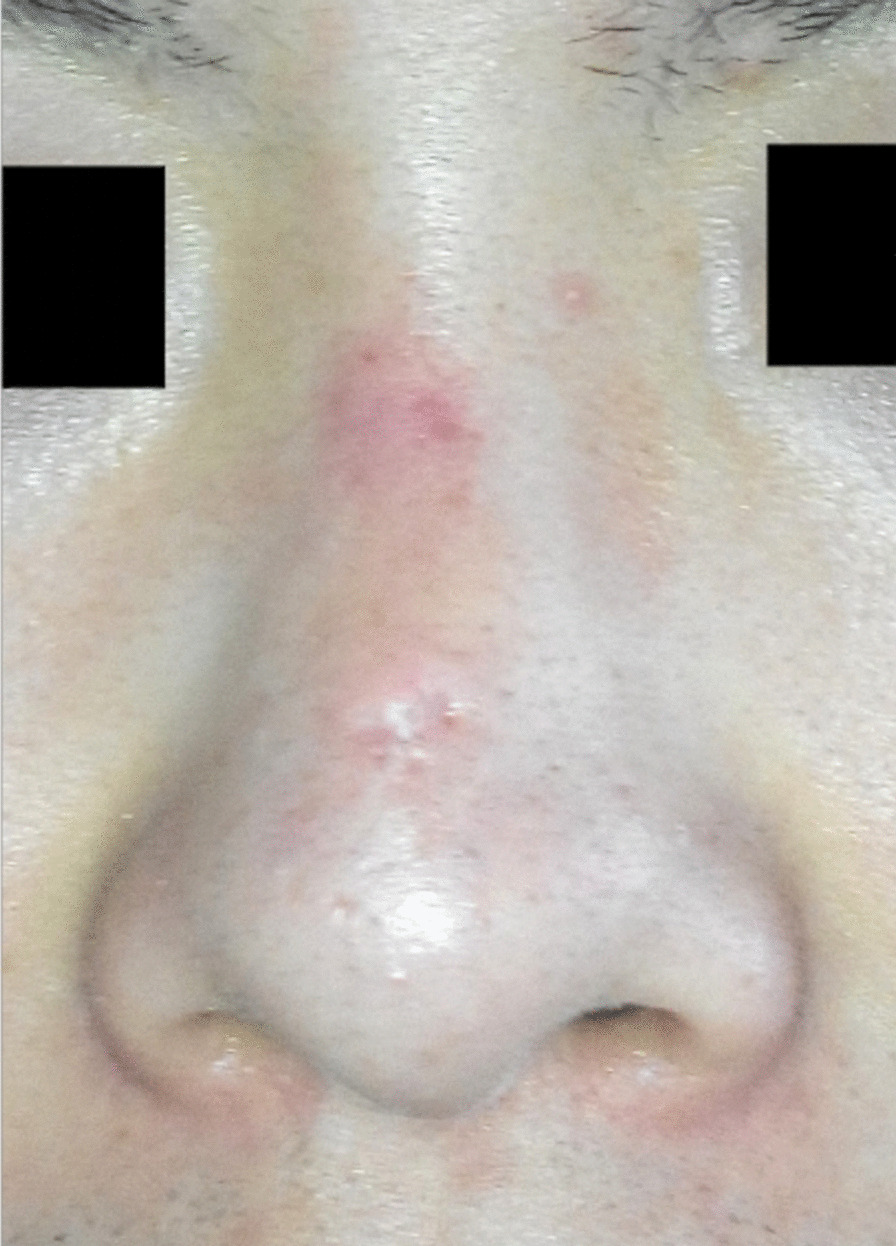
Postoperative picture of the patient(6 months)

**Fig. 7:**
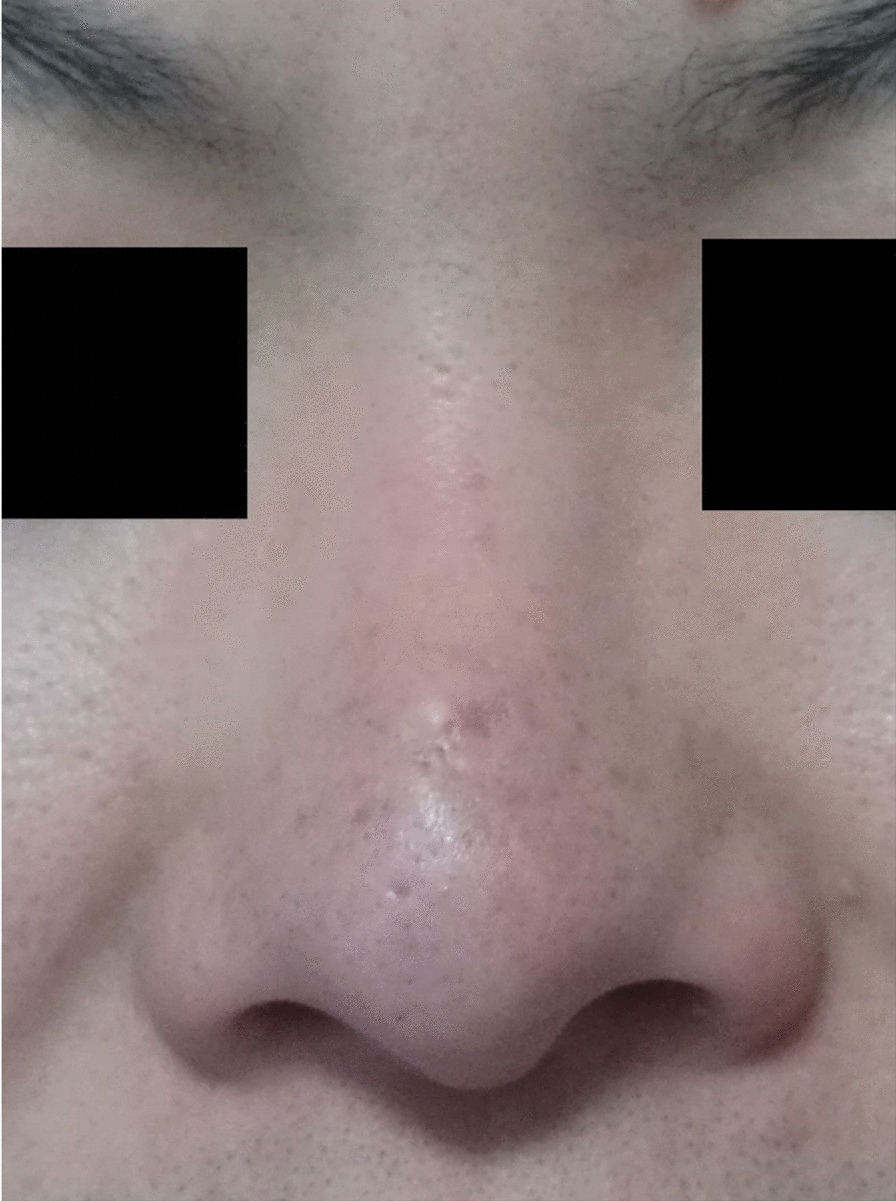
Postoperative picture of the patient (4 years). CT showing the extent of the sinus with in it

The patient’s condition was generally good after the surgery, and the suture was removed on the 7th day, the incision was recovered well, and appearance of the nose was satisfying after half a year (Fig. 6), and even better 4 years later (Fig. 7).

## Discussion

This is an interesting case because the accessory nostril was observed only as a small pit on the nasal dorsum skin which differs in its appearance from all the other previously reported cases. Although, it was successfully treated finally. We still need to know about the multiple appearences of the supernumerary nostril for better treatment.

Supernumerary nostril is a rare congenital defect that defined as the presence of additional nostrils when a pair of normal nostrils already exist, and may be manifested as asymmetric redundancy and differ from complete nasal duplication in horizontal or vertical [2]. The exact etiology is not known, but it is currently accepted to be caused by a fissure of the nasal process during the aberrant embryonic development [3].

Some of the cases are one of the manifestations of the multiple malformations, while others are simple multiple nostrils that show the presence of distinct cavities or localized bulges, totally different from the appearance of a normal nose [4–6]. None of these reports is similar to the case we presented in this article, given that the accessory nostril was observed only as a small pit on the nasal dorsum skin and is not easily detectable. We can infer from this case that differential diagnosis and timely treatment of this disease values a lot.

First of all, pay attention to the personal history of patient, along with the symptom of the disease, as many of the cases are reported to be associated with multiple congenital malformations. Second, imaging examinations such as CT scan are necessary, helping us to exclude the presence of other problems [7]. And if the supernumerary nostril is a clear orifice, it may appear in cross-sectional imaging as a redundant outward projection of the nasal wing, connected to the adjacent nostril by air [7].

This case actually belongs to congenital nasal malformation, which must be distinguished from encephalocele, nasal dermal sinus, nasolacrimal duct obstruction, mid facial cleft and proboscis lateralis [8, 9]. The diagnostic points are the following: (1) Mostly located above the nosewing, which appears like accessory nostrils, with rhinothrix and occasionally white mucus excretions, without breathing; (2) The abnormal nostril is made of skin soft tissue without cartilage, and is not connected to normal nostrils; (3) Auxiliary examination proves that it does not communicate with the nasopharynx. The end of the small hole is blind, or connected with the ethmoid sinus or maxillary sinus, which often accompanied by nasosinusitis; (4) Histology reveals hair follicles, hairy roots and sebaceous glands, but no olfactory cells are found.

Due to its rarity, the exact timing of the intervention and the treatment modality have reached no consensus. Mostly, the literature favors early surgery to ameliorate the medical risks it may pose such as psychosocial disorders or any derangement to the nasal [10, 11]. In our case, although there was no obvious malformation as described in previous literature, it still affected the patient’s appearance to some extent. Meanwhile after closing the sinus opening palliatively, the recurrent postoperative inflammation occurred and made it important to remove the lesion completely by surgery. In our case, although there were no obvious aesthetic defects, recurrent focal infections and unexplained discharge did bring discomfort to the patient’s life. Therefore surgery is a good option. The traditional treatment option is to remove the nasal tract and correct the ala [11]. The challenge of reconstruction is to restore nasal airflow while achieving nasal symmetry. In our case, the redundant nostril was not adjacent to the normal nosewing or normal nostrils and did not inappropriately affect the normal nostrils either in appearance or function. Therefore, fortunately, for this patient, a complete excision of the nasal tract was sufficient. And we opted not to remove all the excess nostril tissue and to excise only the nasal tract, for the skin can be used to cover the soft tissue defects caused by the excess nostril resection and lead to minimal deformation.

## Conclusions

In this study, we presented a concealed case of supernumerary nostril.

manifested as a small pit located on the nasal dorsum in a male adult. We made final diagnosis based on the clinical appearance, physical examination and histological features of the supernumerary nostrils. As described above, the cause and development of this disease is still unclear. However, we advocated early surgical treatment and suggested to handle similar situations with the columellar Inverted-V incisions. Early treatment is recommended to prevent any possible alar or nostrils deformity, as well as avoiding psychological problems.

## Data Availability

Not applicable.
